# Towards poverty alleviation in developing countries: An empirical study of the impact of land tenure reforms in Kati, Mali

**DOI:** 10.1371/journal.pone.0246502

**Published:** 2021-03-04

**Authors:** Brahima Coulibaly, Gideon Sagoe, Li Shixiang

**Affiliations:** 1 Department of Land Management, School of Public Administration, China University of Geosciences, Wuhan, PR China; 2 Department of Land Management, University of Ségou, Ségou, Mali; 3 Department of Process Engineering, Sewerage Systems Ghana Limited, Accra, Ghana; Wroclaw University of Economics and Business, POLAND

## Abstract

Post-colonial land tenure reforms in emerging countries have partly aimed at poverty reduction through equitable land access. However, the poverty rate keeps rising in rural and peri-urban settings in Sub-Saharan Africa dominated by agricultural activities. This article reviews land tenure reforms in Mali, from the year 2000 to 2017 regarding poverty alleviation and evaluates their impacts on indigenous smallholder farmers, using multiple linear and logistic regression models and local experts’ elicitations. The results indicate that the advent of land titles as the only definitive evidence of land ownership, following the reforms, has generally weakened customary land management. Smallholder farmers face several barriers to obtaining land titles, limiting equity in land access and security. This has paved way for land markets marred by irregularities and resulted in colossal loss of agricultural lands, which are the main source of rural livelihood. Thus, the reforms have not yielded the intended poverty reduction outcomes. The study recommends that land transfers must be authorised by a single institution, represented at the various administrative levels, which issues an authentic and incorruptible document using appropriate technology. Moreover, since pro-poor provisions in the reforms usually lack implementing decrees in Mali, political will is key to achieving equitable land access and security.

## Introduction

### Background

Land tenure and governance are critical factors in the eradication of hunger and poverty and contribute to the sustainable use of the environment [1,2]. They play a central role in determining whether individuals, communities and other actors can acquire the right to use and control natural resources, such as land, under specified conditions over a period. Each society defines and regulates how individuals, communities and entities access land and other common goods through its tenure systems. Generally, land tenure systems may be based on either written policies and laws or unwritten customs and practices. The issue of land tenure is particularly important because the livelihoods of many people, especially the poor in rural areas, depend on the access and control of land and other natural resources. These resources do not only serve as the source of food and shelter, but also as the basis of their social, cultural and religious practices, and are pivotal in economic growth [3].

Equitable land access and security stimulate economic development through land investment, agricultural production, sustainable management of natural resources, development of market economies and even income generation from taxation [4,5]. This has been observed in developed countries with balanced and innovative land distribution reform policies. Conversely, in many emerging economies, access to land by the poor is limited. The limited rights of access to land and other natural resources and the insecurity of these rights often lead to extreme poverty and hunger. As a result, the poorest persons in the world are those that do not have access to land ownership and property rights [6].

In developing countries, one common challenge with land access and security is land tenure dualism. Land tenure dualism refers to the juxtaposition of customary laws and modernistic land-management laws [7] and is at the root of the widespread land disputes in Sub-Saharan African nations, such as Mali [1,8]. Moreover, it partly explains the high pressure of land demand that the urban environment exerts on the rural environment. In Ghana, the three major categories of land tenure (i.e. lineage, leasehold and freehold) have a continuum of land security with different impacts on investment in land conservation [9,10]. Sewornu et al. [11] also described this situation in Ghana as a typical legal pluralism, whereby there is the coexistence of customary laws, rules and statutory laws in a complex mix with the land administration institutions and the associated challenges that arise. In China, Li et al. [12] posited that the dual land tenure system limits the sustainable development of rural China, which is undergoing rapid depopulation and abandonment and inefficient use of land. This dual ownership delimits two land regimes in general; an urban system based on State ownership and a rural system based on collective ownership. It has been reported that this duality of land tenure is one of the most apparent causes of land insecurity problems in modern China [13]. Additionally, the plurality of land tenure is generally a source of land speculation in Africa, where a lack of transparency characterises the land market. As a result, other developing countries, like Vietnam and Kenya, have transitioned to legal singularity, although there are still some challenges, especially in Kenya [10]. Thus, analysis of land markets in Africa generally revolves around how customary land tenure regimes evolve towards private land ownership [4,14].

Usually, land tenure reforms appear necessary to meet the many challenges facing rural people, but only on paper in most cases. For example, in 2004, the South African government passed the Community Land Rights Act, which aimed to secure the land rights of communities and people occupying lands known as community lands that the apartheid regime had reserved for Africans [15]. However, in some cases, the reforms have led to the “capture by elites” and the marginalisation of the poor and minorities. Inefficient and corrupt bureaucracies and the high costs of conventional land titles have also rationed poor and vulnerable groups and favoured the rich [16]. That notwithstanding, in China, a reform on the agricultural land was initiated at the village level, with the Household Responsibility System (HRS), which allowed the allocation or reallocation of lands to individuals in collaboration with the central government and local authorities [17]. As a result, a study showed that from 1978 to 1984, the HRS led to a 7.7% annual rise in agricultural production with a 14% increase in farmers’ income per annum [18].

### Land tenure reforms and poverty alleviation

The evolutionary theory of land rights depicts the gradual shift from customary landholdings towards private, individual and family property rights, driven by population growth and market integration [19]. Land reforms are the vehicle for the transition from customary to private property rights. Notably, the shift has been accompanied by the demand for formal documentation or land titles to secure private property ownership [19]. Particularly in Africa, where customary land tenure system is dominant, land titles have become a cornerstone of land reforms. The use of land title is explained by the guarantee of the right it provides [20], due to the many transparency and reliability measures supposed to accompany its creation. However, the latter is not always the case in some developing countries. For example, in Cameroon, a land title is the only legal means of claiming land ownership. But, while the land title acquisition eliminates gender discrimination in land ownership, the process is often unknown by the masses and excludes the poor [21].

Feder et al.’s conceptual framework for land security and agricultural productivity establishes a positive nexus between land title and economic performance [22]. And after independence, several Sub-Saharan African governments have adopted policies and programs aimed at increasing land security for farmers to promote agricultural investment and productivity. Conversely, generally, the current legal frameworks in the sub-region have several gaps. For example, in these frameworks, there is either a total non-recognition of customary land rights [21] or existing customary land management practices remain a vector of inequalities and exclusions against the vulnerable [23]. Since the lack of access to land and tenure insecurity are closely linked to poverty and inequality [24], these gaps in the land laws adversely impact on the socio-economic and cultural conditions of the people. Therefore, policy reforms are quintessential to improve global governance of land resources [21].

Since the 1980s, land reforms in many developing countries have integrated elements of poverty alleviation. International institutions, such as the International Monetary Fund (IMF) and the World Bank [25,26] have supported these efforts. For example, the World Bank supported the government of Ghana to carry out land reforms in 2003, through the US$ 55 million Land Administration Project (LAP), which aimed to increase access to land titles, strengthen community-level registration institutions, reduce boundary disputes and harmonise customary and statutory laws [25,27]. Despite the joint efforts of these international bodies and local stakeholders, including the government and civil society organisations, there is a persistent increasing trend of the poverty rate. As noted earlier, equitable access to natural resources, particularly productive lands, can serve as a better solution to alleviate poverty. However, regardless of the various reforms over the years, land access and security remain problematic in rural areas, worsening the poverty crisis.

Although recent reforms recognise customary land rights, in practice, there are still gaps in their materialisation to secure lands, especially for subsistence farmers. This is because usually, the laws that recognise customary rights also provide definite ownership to land title holders. As a result, economists and statisticians are not unanimous on the issue of land law and its influence on investment and agricultural productivity [28]. To some scholars, the link between land law, investment and agricultural productivity is positive [4,10]. However, others disagree. For example, in Ethiopia, Tura [29] reported that pastoralists and smallholder farmers do not benefit adequately from statutory protection. Instead, the laws and practices enable arbitrary evictions and land grabbing, leading to the economic marginalisation of most ethnic groups. Another study [30] conducted in Tanzania also found no significant impact of land titles on agricultural production and investments. Therefore, there is a need to undertake massive reforms that tackle land access and security inequalities. Agrarian land reforms should be pursued in broader frameworks of development initiatives, and these frameworks must be comprehensive and integrated to function proactively for the poor [31]. They should identify and target appropriate sub-groups and apply appropriate strategies to achieve the intended poverty alleviation outcomes [32]. Moreover, some scholars say that the implementation of legal regulations to the customary land tenure and participation of local citizens in the planning process are key to successful land use planning and reforms [33].

### Objectives of the study

Although there are several studies on land tenure reforms in Mali, relatively little attention has been given to the effects of the reforms on the poor, especially in peri-urban and rural settlements. This paper analyses the impacts of the land reforms in providing appropriate solutions to land management issues in Mali from 2000 to 2017, focussing on poverty reduction. First, we present a non-systematic review of the reforms. Next, using the Kati *Cercle* (third-level administrative division in Mali), in the Koulikoro region, as a case study and expert elicitations, we (1) assessed the poverty level among smallholder farmers based on annual income; (2) investigated the main factors that influence yearly income and; (3) identified some of the critical implementation bottlenecks of the pro-poor provisions of the land reforms in the country.

### Review of reforms and improvements of land tenure for poverty alleviation in Mali

Mali, like many countries in the West African sub-region, faces multiple problems in land governance. Since 70% of the country’s 18.1 million population lives in rural areas [34], there is increasing pressure on lands and conflicts that affect land access and food security. Also, land management problems straddle in different sections of the populace because agro-pastoral activities represent the leading economic sector and employ more than two-thirds of the population [34]. The intense pressure on land is also linked to the global population evolution, climate change, the loss of soil fertility and the need for biofuel [1,8,9].

Post-colonial land tenure reforms in Mali started with the Federal Land Adoption Code of 1963 (*Code Domanial*). Later, the Land Code of 1986 was enacted. Article 3 section 1 of this Code gave full ownership of all lands in Mali to the government but acknowledged private property rights only when the owner has a land title deed. Although it recognised customary land rights, such rights were deemed subservient to land titles, raising land insecurity issues for non-statutory holdings [20]. Subsequently, land legislation in Mali has been revised several times since the early 1990s. These reforms aim to harmonise land legislations with the liberal economic policies implemented, following the structural adjustment plans, which introduced the private property rights, and the March 1991 democratic transition that abolished the 1991 Forestry Code. This period was the starting point of major political, economic, and social reforms. Since then, there has been a coevolution of land policies, political changes, and global economic policies, referred to as the “global/sectoral connection,” by some scholars [14].

Contemporary land reforms in Mali started in the year 2000, with the *Code Domanial et Foncier* (CDF) or the 2000 Land Tenure Code [1] and the creation of the Ministry of State and Land Affairs (MDEAF). In 2006, the *Loi d’Orientation Agricole* (LOA) or the 2006 Agricultural Framework Law was established by the National Assembly. A National Consultation on Land reform became imperative, due to the prevailing challenges, and was conducted in 2008–2009 to revise the CDF 2000. This dialogue led to the most recent reform, the *Loi Foncière Agricole* (LFA) or Agricultural Land Law, in 2017.

### Land Tenure Code 2000 (CDF 2000)

The CDF 2000 considered the decentralisation and democratisation in Mali, their influence on land issues and also the need for bold and consensual reform. The first stage was the issuance of land titles, which had already been the subject of many institutional efforts [35]. Also, it provided that state and non-state actors must recognise that land, forests and fisheries have cultural, spiritual, social, economic, environmental and political values to indigenous people and other communities with customary land regimes [3]. Furthermore, it emphasised that land transactions based on customary practices and procedures could be formalised in writing to serve as proof [1]. Although the CDF 2000 strengthened private ownership of land, it had similar challenges as the Land Code of 1986 and, thus, could not resolve the issue of inequitable land access and security. For example, Section 1 of Article 6 of the Code juxtaposed the two types of rights: a customary law establishing family or customary ownership of land, and a modern law based on deeds of property issued by the administration. However, the aspects that dealt with respecting customary rights were never translated into decrees and administrative practices [1].

In recent years, in line with the 2005 Strategic Framework for Combating Poverty (CSLP), the government of Mali has expressed its intention to prioritise combating poverty through its development activities. Achieving the CSLP’s objectives largely depends on the performance of the agricultural sector. To this end, some land reforms were instituted to provide better land security for farmers and increase agricultural production and the income per family farm. The two main reforms are the LOA 2006 and the LFA 2017.

### Agricultural Framework Law 2006 (LOA 2006)

The LOA was established in September 2006. Former President, Amadou Toumani Touré, considered this legislation as a powerful tool to enhance the national agricultural potentialities and was actively involved in its enactment. The LOA 2006 had the following specific objectives:

- Issuing various certificates for agricultural land transactions to secure individual or collective land rights, and family farm rights;
- Establishing a coherent land management framework at the national level while taking into account the decentralisation;
- Promoting and supporting the land management institutions, particularly, land commissions in the view to strengthen the involvement of rural populations in agricultural land management.

Primarily, the LOA 2006 presented a framework of Mali’s agricultural sector and provided guidelines on how later implementing decrees will structure land tenure systems on agricultural lands [26]. Additionally, it acknowledged the value of both statutory and customary land rights. It also aimed at reducing the cost and procedural complexities associated with securing land titles and rural concessions, for equitable access and security of lands for agriculture [1,26]. Article 83 states that priority must be given to vulnerable populations (women and the youth) in the allocation of irrigated agricultural lands. It also introduced the creation of *commissions foncières* (land commissions) at the regional and commune levels. The commissions’ responsibilities included resolving land conflicts before they escalate to the law court, compiling and mapping traditional practices relating to land tenure, and developing local cadastres [26]. Subsequently, in January 2009, a decree was passed to institute the land commissions and its membership composition. A land commission is chaired by the Prefect of the *Cercle* (or their representative), and the decree specifies other members, including land chiefs in the jurisdiction. However, at the local level, nominations are done by the Prefect. Additionally, the reform had some geopolitical implications, such as the decentralisation in Mali and the integration of sub-regional policies.

Despite these reforms and recommendations over the period, land management in Mali was still a thorny issue, which drew much public criticism due to arbitrary expropriations, sometimes bloody land disputes, the diversity of land allocation documents issued by agents, and institutions of overlapping mandates, and sometimes contradictory court decisions. For instance, on Oct 22, 2014, the Council of Ministers noted that the scale and depth and sometimes the violence of land conflicts registered in the country highlighted the importance and sensitivity of land problems both in rural and urban areas. The number of land-related cases kept rising in the law courts, which were already overwhelmed by the high number of complex land disputes. For example, during the Forum for Democratic Discussion held on Dec 08, 2014, land disputes constituted the overwhelming majority of interpellations. Similarly, the round table discussion devoted to land reform in October 2014 found that more than 70% of law cases concerned land disputes [36].

### Agricultural Land Law 2017 (LFA 2017)

Owing to the above challenges, the government launched the *Etats Généraux du Foncier* (National Consultation on Land) to revise the CDF 2000. The dialogues were held at the *Cercle* and regional levels, and among professional bodies at the national level. The key recommendations from the consultations included the need for the government to (1) establish an explicit and transparent regime for the management of irrigated farmlands; (2) clarify the place of local conventions in land and natural resource management; and (3) provide a suitable approach for raising awareness of the content of the law [1,37]. It also proposed the adoption of measures facilitating women’s access to land, the revision of the Mining Code, the entire delimitation of the land reserves, farm registration, and the strict enforcement of breaches. Other cross-cutting recommendations included the establishment of an observatory of land and real estate, digitisation of the land register with regular register updates and the registration of reserved or designated public lands [1,37].

Based on the recommendations of the National Consultation on Lands, a project for a new land law was proposed. After its proposition in June and approval in July 2014, the project went through several modifications. In October 2015, the original version was decreed by the Council of Ministers and adopted by the National Assembly. However, farmers’ associations, as well as some civil society groups vehemently opposed it. They argued that the law had inherent inconsistencies that constituted a threat to the right of housing and family farming; it also lacked a clear and effective system of implementation for land conflict resolutions, which was critical for the country’s stability.

After revisions, the LFA 2017 was finally enacted in April 2017. This law aims to enable the materialisation of the customary land rights and to translate the main legal guidelines of the Agricultural Land Policy (PFA) based on the following objectives:

- True recognition of the customary land rights of an individual or group through a certificate of customary detention issued by the village chief and authenticated by the Mayor of the Municipality, and also through a certificate of land ownership issued by the Mayor;
- Recognising and securing of a large panel of land transactions through certificates issued by the village chief;
- Creating municipal registers of landholdings and municipal registers of land transactions;
- Establishing village authorities through land commissions, and the issue of land certificates, and the social judgment of agricultural land-related conflicts.

Generally, the LFA 2017 deepens the LOA 2006 and contains significant innovations, including the recognition of the prevalence of customary rights and local land management. Besides, this law recognises the collective customary land rights of families and village communities. Also, because land management is linked to the customs and traditions of various communities, the LFA 2017 adds that a “mapping of customs and traditions” should be carried out at the community level. Moreover, it stipulates that 15% of agricultural land must be allocated for so-called “vulnerable” groups, women and young people.

## Materials and methods

### Context of the case study area

**Fig 1:**
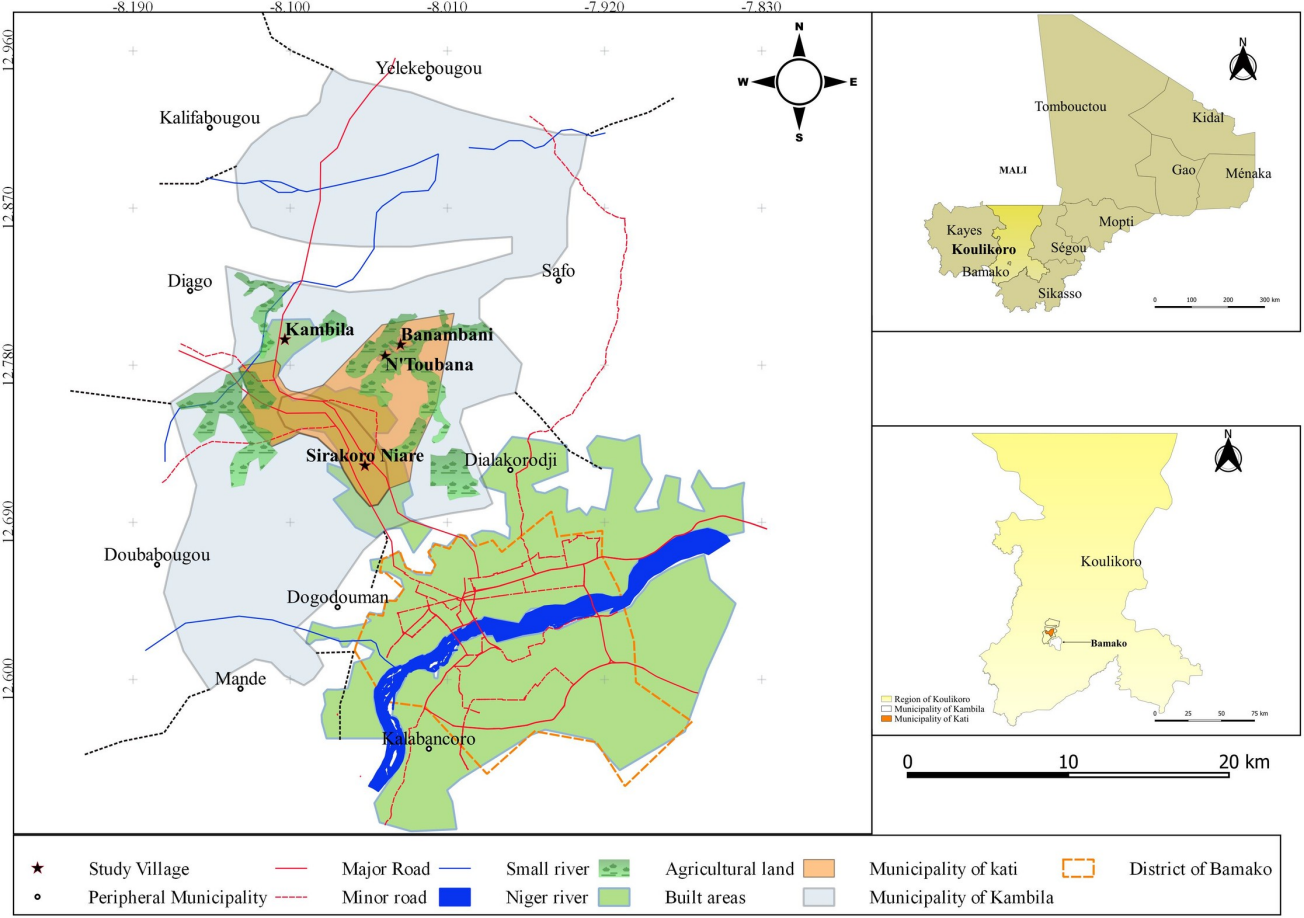
Map of the study area.

This study was conducted in the urban Commune of Kati and the rural municipality of Kambila, both in the Koulikoro region of Mali (see Fig 1). Kati was chosen for this study because it is known to be a hotspot region of acute land security problems in Mali. In 2015, for example, the head of Domain and Cadastre bemoaned the high decreasing rate of cropland areas in Kati and the several factors that complicate land tenure security in the area [38].

The *Cercle* of Kati, which includes both the Commune of Kati and the rural municipality of Kambila, has a population of approximately 120,000 inhabitants. Similar to the national population structure [34], Kati has more females (51%) than males (49%) [39]. The average household size is 6 persons, though household size can be as high as 18 persons in some neighbourhoods. The population of Kati is composed mainly of the Bambara ethnic group. However, some ethnic minorities such as the Peulhs, the Malinkes and the Sarakholes are represented [34]. Kati’s main economic activity is agriculture, mainly millet and sorghum cultivation. However, subsistence farming is widely practised due to poor rainfall, soil infertility and the use of rudimentary equipment and technologies. Like most rural areas of the country, pastoralism coexists with agriculture. It holds an important place in the family economy and constitutes a source of income for the population. The youth population mostly migrate to the capital city, gold mining sites, neighbouring countries and Europe for greener pastures.

### Data collection

Four villages were chosen for the study, namely, N’Toubana, Banambani, Sirakoro-Niare and Kambila. These places were selected because, unlike other surrounding villages, they are currently under enormous land pressure due to their proximity (about 25 km radius) to Bamako, the capital city of Mali, which currently has no land available for expansion. As a result, there are serious land conflicts between the inhabitants of these four villages and real estate companies, which have attracted national attention [38].

We used both quantitative and qualitative techniques to elicit information from smallholder farmers and local experts, respectively, to evaluate the impact of the land tenure reforms in the study area, and Mali by extension, from the year 2000 to 2017. The data was collected in January and February 2018. Due to security reasons in the area, most of the farmers in the study communities were reluctant to participate in the study. As a result, we limited our sample size to only 100 smallholder farmers (all household heads). We interviewed 25 farmers each from the selected villages, using a semi-structured questionnaire (see S1 File). Four (4) opinion leaders (i.e. the chiefs of N’Toubana, Banambani, Sirakoro Naire from Kati, and Kambila) were part of the 100 respondents. Moreover, three key informants were interviewed to validate and give further insight into the findings from the survey. They were the General Secretary of the UACDDDD (Union des Associations et Coordination d’associations pour le Développement et la Défense des Droits des Démunis or Union of Associations and Coordination of associations for the Development and Defence of the rights of the poor); the General Secretary of the MSV (Mouvement des Sans Voix or Movement of the Voiceless); and Lawyer Bourama Sagara, highly recognised for his expertise in land issues in the country. The UACDDDD is the mother umbrella body of about 213 member associations, which are at the forefront, fighting against various forms of social injustice in the country. Similarly, the MSV is also popular, inter alia, in land rights advocacy for the poor and marginalised.

### Ethics statement

The method used in this study was approved by the Steering Committee of the Centre of Expertise and Applied Research for Development (CERAD) of the University of Ségou, Mali. The study involved the participation of humans, as respondents, in a survey and interviews. There were two groups of respondents, (1) smallholder farmers and (2) three experts. The study was voluntary and verbal permissions were sought before the participation of any of the respondents. The respondents in Group 1 were assured of the confidentiality of the information they provided. Accordingly, all responses, including the respondents’ demographic data, were reported in aggregate or in anonymity to eliminate potential inappropriate use of the information. Moreover, the questions asked were clearly explained to the respondents in their local dialect to be able to obtain their true responses as much as practicable. On the other hand, prior to the interviews, participants in Group 2 were made aware that their names may be published along with their comments, and all three experts to this condition before the interviews.

### Theoretical framework and definition of variables

Theoretically, land reforms can be used as a tool for equitable land access and security to ensure food security and reduce poverty. Therefore, since agriculture is the main economic activity in the study area, poverty level among smallholder farmers could be used to evaluate how land reforms, over the years, have successfully yielded poverty reduction outcomes in Mali. Poverty can be defined in several ways, such as the highly-criticised welfarist approach (relates poverty to individual utility levels); the capability approach (links poverty to nutrition, health and education); and the basic needs approach (links poverty to access to nutrition, primary health care, basic education and sanitation) [32]. The latter approach is usually applied in developing countries, with the construction of poverty lines based on an aggregation of the variables [32]. As such, this study evaluated the poverty level of the farmers by comparing the estimated annual income with Mali’s poverty line stated at FCFA 177,000 (303.1 USD) [34].

The annual income of farmers primarily depends on the sale of produce from their farms. However, income (as a dependent variable) may be influenced by variables such as age, family size, level of education, occupation, and land size. Age may affect income because old-aged smallholder farmers may not have the strength to cultivate their lands. In the traditional setting, larger families imply more labour force to work on family farms and vice versa, and could, therefore, predict family income [40]. Farmers may have multiple sources of income if they have other occupations aside from farming. Level of education may predict family income [41], as a higher educational level may increase the chance of a farmer having an extra job for extra income, and can also contribute to the proper management of farm for optimal benefits. The size of agrarian land presently cultivated and the percentage decline in farmland size relate to the total annual agricultural output, which in turn can be an indicator of yearly income [32]. Also, farmers may earn some income from the sale of land, but the price per hectare could significantly predict their income levels.

Furthermore, smallholder farmers may lose their agricultural lands to voluntary or involuntary land sales, or as a result of compulsory acquisition by the State or despoilment. This study investigated the likelihood of a farmer being a victim of land expropriation or despoliation. The predictor variables included annual family income, educational level, size of land owned in the past, the presence of land chiefs, farmers’ opinion on the impact of urbanisation on customary landholding right, their perception of the justice system in settling land disputes and their awareness of other villagers being dispossessed of their lands. We hypothesised that family income might determine their financial capacity to register their lands and also employ the legal process to protect their lands as compared to customary land rights; hence, it can influence the likelihood of land loss. Education level will also correlate with knowledge of farmers’ rights and their ability to protect their farmlands from despoliation [42]. Land registration (in the form of village attestation, award letter, provisional title or full land title) offers better land security as compared to customary rights. Thus, as compared to customary right holders, it will be less likely for farmers to be deprived of access to their registered lands. Similar to the tradition in some Sub-Saharan African countries [26,43], land chiefs (*chefs de terre*) in Mali are agents of land dispute resolution. A land chief is the head of the descendants of the founding ancestor of the village (or the first family to settle in an area). According to tradition, all the village lands belong to the “*chef de terre*,” who can donate, lend or give as gifts to non-natives who want to settle in the village. They are legally recognised by the State as representatives of customary authorities, and provide the primary witness to customary land transactions. Hence, it was hypothesised that the presence of a land chief could reduce land loss, especially through despoliation. Moreover, not only does urbanisation promote the loss of agricultural lands (e.g. due to youth emigration and low-yield of land) [44]. It also adversely affects customary rights [7], increasing the likelihood of farmers being dispossessed of their farmlands. Moreover, farmers’ awareness of other villagers losing their lands to expropriation or despoliation may suggest the prevalence of land dispossession in the area, increasing the possibility of the respondent also being a victim. Lastly, it was assumed that farmers who lose their lands might seek redress at the law courts, and fairness of Mali’s justice system could reduce the likelihood that farmers are unlawfully evicted from their lands. Thus, a farmer’s perception of the justice system of Mali in land disputes settlement may predict the possibility of being victims of despoilment.

### Data analysis

The data obtained using the questionnaire were organised and analysed using version 24 of the Statistical Package for Social Scientists (SPSS) software. The codings of the variables are presented in the S1 Table. A multiple linear regression model (Eq 1) was used to determine how some selected variables predicted the farmers’ yearly income. To minimize possible sampling biases due to the relatively small sample size (*n* = 100), the bootstrapping technique (*N* = 10,000 at 95% confidence interval) was applied [45].

$$Y=\alpha +\beta_{1}x_{1}+\beta_{2}x_{2}+\ldots +\beta_{8}x_{8}+\varepsilon$$ (1)

Where *Y* indicates the respondent’s annual income; *x*_1_ represents age, *x*_2_ is occupation, *x*_3_ is level of education; *x*_4_ means family size; *x*_5_ indicates the size of land presently owned; *x*_6_ is the % decline in farmland size; *x*_7_ denotes the value of land sold (CFA Franc per hectare); *x*_8_ is the total tonnage of annual crop production. Also, α denotes the constant, *β*_1_, *β*_2_,…β_8_ indicate the respective coefficients of the independent variables, whereas ε is the error term in the model.

Additionally, using a logistic regression model, described by Eq 2, we examined how various variables influenced the likelihood of a farmer being a victim of land spoliation or expropriation. All the dependent variables were entered into the equation.

$$ln\left(PX/(1-PX)\right)=\beta_{0}+\beta_{1}X_{1i}+\beta_{2}X_{2i}+\ldots +\beta_{k}X_{ki}$$ (2)

Where the subscript *i* is the *i*th observation in the sample; *PX* is the probability of an event occurring for an observed set of variables *x*_1*i*_; (1 –*PX*) is the probability of not being a victim of land despoliation. *β*_0_ is the intercept term and *β*_1_, *β*_2_…*β*_k_ are the coefficients of the explanatory variables *X*_1_, *X*_2_… *X*_*k*_, respectively.

In both models, the variance inflation factor (VIF) and tolerance values were computed to evaluate the level of multicollinearity among the predictor variables [45]. Also, where applicable, we explored the relationship between variables by computing their correlation coefficients and assessing the levels of significance.

## Results

### Demographic characteristics of the respondents

All the respondents were males and 90% of them were married. The average age of the respondents was 54.6±1.4 years, with the majority (94%) of them aged 35 years and above (Table 1). Almost a quarter of them had no formal education and were illiterates, with an equal percentage having basic education, while 4% and 8% had secondary and tertiary education, respectively. A significant proportion (30%) were literates but through adult education programmes. The average household size was 14.0±0.4, which is higher than the average for Kati. Moreover, 47% of the respondents had no other occupation, aside from agriculture.

**Table 1:** Demographic characteristics of respondents.

	Category	Percentage (%)	Average (SD)
Age (yr)	25–34	6	54.6 (1.4)
	35–44	23	
	45–54	28	
	≥ 55	43	
Level of education	None	24	
	Literates (adult education)	30	
	Basic (1^st^ or 2^nd^ cycle)	24	
	Secondary	4	
	Tertiary (diploma or degree)	18	
Occupation	Agriculture only	47	
	Self-employed	31	
	Private company	17	
	Public service	5	
Family size	5–10	28	14.0 (0.4)
	11–16	34	
	≥ 17	38	
Annual family income (CFA Franc)	< 300,000	11	603,000 (93,899)
	3000,000–599,999	42	
	6000,000–899,999	36	
	≥ 9000,000	11	

### Poverty among smallholder farmers in Kati

The average annual family income was 603,000±93,899 FCFA (1,097.5±170.9 USD). Based on the average household size obtained in this study, we estimated the average annual income per capita at FCFA 43,071 (78.4 USD), which is far less than the national poverty line of FCFA 177,000 (303.1 USD) [34]. Table 2 presents the results of the multiple linear regression model, which shows the effects of the explanatory variables on family income. The model’s adjusted *R*^2^ value was 0.235, with relatively insignificant multicollinearity among the predictors (average VIF = 1.339 and Tolerance > 0.2) [45]. The results indicate that farmers’ annual family income is significantly influenced by the percentage decline in the size of their farmlands and the amount they earned from land sale per hectare. The value of land sold (per hectare) positively predicted farmers’ income (*β* = 0.607, *p* < 0.01). Also, the percentage decline in farm size (*β* = -0.453, *p* < 0.05) had a statistically significant negative effect on farmers’ annual income, suggesting that farmers who have lost larger proportions of land have lesser income. However, the predictive effects of the respondents’ age, occupation, educational level, household size and the annual farm productivity (in tonnes) were not statistically significant.

**Table 2:** Effects of selected variables of farmers’ annual income.

Independent variable	Coefficient		*t*	*p*-value	95% CI for *β*		Collinearity Stats.	
	*β*	Std. Error			LB	UB	Tolerance	VIF
Age	.335	.243	1.381	.171	-.147	.817	.838	1.193
Occupation	.658	.508	1.295	.203	-.351	1.667	.687	1.456
Level of education	.217	.146	1.490	.165	-.072	.507	.616	1.624
Family size	.212	.326	.835	.460	-.376	.921	.637	1.570
Land size (current)	.024	.305	.091	.926	-.579	.635	.808	1.237
% decline in farmland size	-.453**	.187	-2.456	.031	-.833	-.088	.868	1.153
Income from the sale of land per hectare	.607**	.163	3.578	.001	.259	.905	.770	1.298
Annual tonnage of crop produced	-.220	.243	-1.093	.249	-.749	.217	.847	1.181
(Constant)	2.603	1.473	1.767	.081	-.323	5.530	-	-

The dependent variable is annual income; bootstrapped sample size, *N* = 10,000; Model’s *R*^2^_(Adjusted)_ = 0.235, *F* = 4.795, *p* < .001.

** denotes that the coefficient is significant at 5% level.

### Land access and security in Kati

From Table 3, the mode of acquiring land in the study area is primarily by inheritance (56%), followed by purchase (19%), lending or as gift (16%), rent (5%) and from the Town Hall (or urban municipal authority) (4%). Most (53%) of the respondents had a mixture of customary and modern land tenure rights to their plots of land, while 38% had only customary rights, and 9% with only the modern land tenure rights. The majority (66%) of the respondents said that women have access to land, with the remainder disagreeing. Moreover, only 29% had registered their entire plots of land; 6% had full titles, 6% held provisional land titles, 13% with award letters and 4% with village attestation. Land registration correlated positively with respondents’ level of education (*R* = .261, *p* < 0.05). Most of the farmers cited the long and complicated procedure of obtaining land titles (56%) and financial constraints (31%) as the main reasons they had not registered their lands. It is, therefore, not surprising that 64% of the farmers favoured the idea that dualism of the land tenure in Mali, could help alleviate poverty. Also, 69% of the respondents admitted that the authorities currently recognise customary land rights, especially after the Agricultural Land Law 2017 was enacted.

**Table 3:** Percentage distribution of respondents’ responses to various land issues items.

Items	*n*	Category	Percentage (%)
Mode of land access	100	Intra-lineage	56
		Purchase	19
		Town Council (Municipal Authority)	4
		Gift or lend	16
		Rent	5
Land tenure type	100	Customary	38
		Modern	9
		Mixed	53
Land(s) registered	100	Yes	29
		No	71
Reason for not registering your land	71	Lack of financial resources	31
		A long and complicated procedure	56
		No idea	13
Perception about the modern land law (LFA 2017)	100	Good	69
		Bad	31
There is justice in land dispute settlements	100	Yes	64
		No	36
Dualism of land tenure alleviates poverty	100	Yes	64
		No	36
Reason for land sale	100	Family needs	29
		Fear of losing to the government	41
		Purchase farm equipment	26
		Other	4
Land buyer	100	Real estate companies	3
		Rich traders	34
		Individuals	59
		More than one option	4
Reason for land purchase	100	Housing	30
		Agriculture	20
		Breeding	7
		Land speculation	37
		Other	4
		More than one option	2
Land sale/purchase middlemen	100	Agent	9
		Professional surveyor	13
		Native	29
		Land speculator	44
		Bailiff	1
		More than one option	4

### Drivers of agricultural land loss in Kati

**Fig 2:**
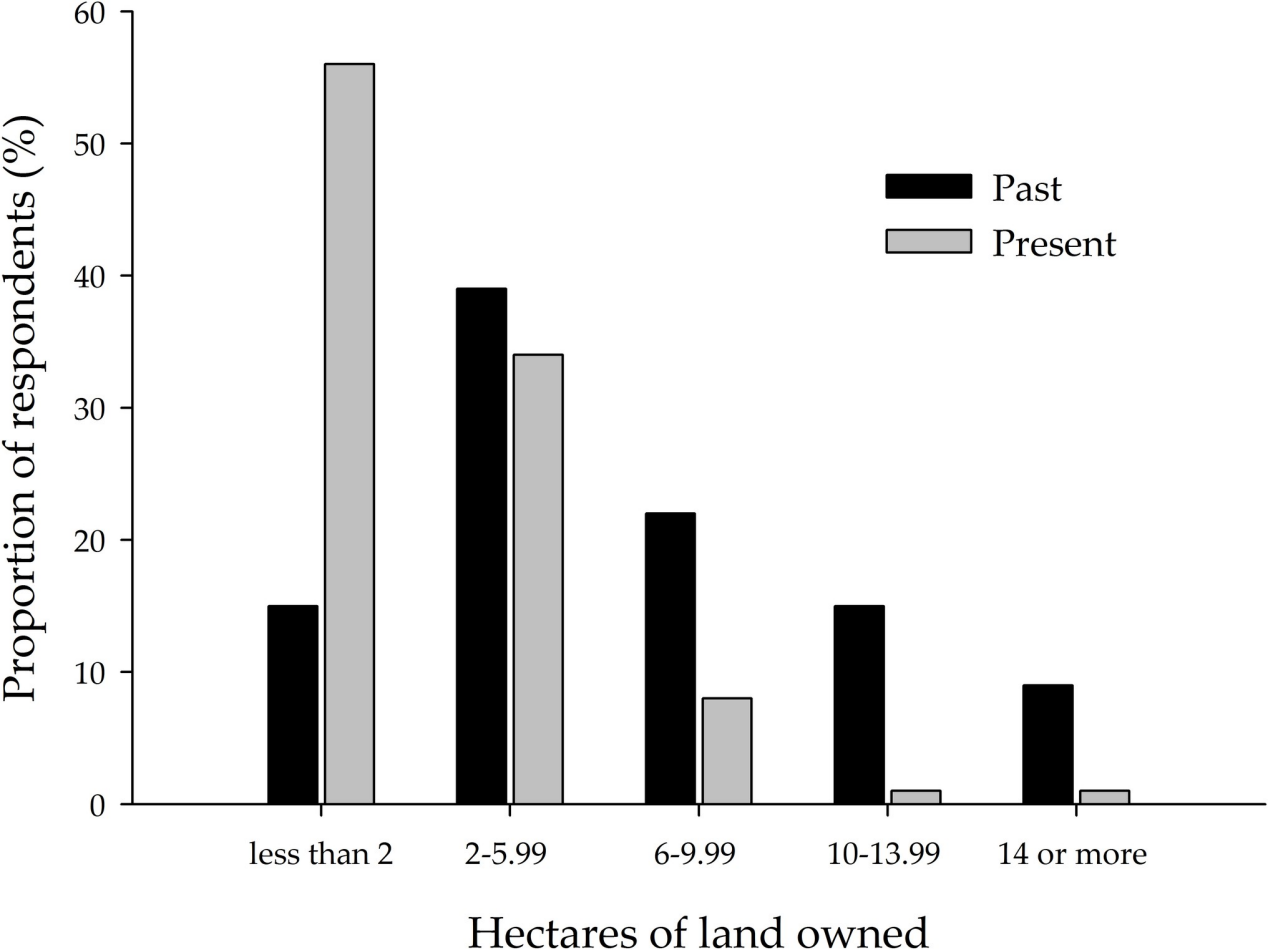
Size of land owned by farmers in the past and at present.

The survey data shows that 58% of the respondents had lost at least 60% of their lands, with one-third losing 20–59%. Fig 2 illustrates further the decline in farmers’ land size. The majority of the farmers possessed 2–5.99 ha of land in the past. However, most of them currently own less than 2 ha. At present, 56% of the respondents owned less than 2 ha land, as compared to 15% in the past. This observation indicates the loss of lands belonging to the farmers. There existed a weak but statistically significant positive correlation between present land size and the total yearly tonnage of agricultural production (*R* = .320, *p* < .05). Hence, at the time of the study, the entire farm output of the respondents was mostly 0–2.2 tonnes (52%) and 2.3–4.5 tonnes (32%). Additionally, the percentage decline in farmlands positively correlated with family size (*R* = .305, *p* < .05) but was not significantly related to the level of education.

The findings of the survey indicate the loss of agrarian lands owned by smallholder farmers in Kati (Fig 2), and its significant contribution to the prevalence of poverty (low income) among the rural farmers (see Table 2). Agrarian land loss was mainly attributed to land sales and despoliation (including expropriation), among other causes. Over a quarter (27%) of the respondents were victims of despoliation or expropriation. Also, the majority (41%) of the farmers had sold portions of their land for fear of losing it to the government, while 29% and 26% of them sold it to cater for family needs and buy farm equipment, respectively (Table 3). Moreover, the driving forces of land purchases from the farmers were primarily land speculation (37%), housing (30%) and agriculture (20%). In most cases, the lands are bought by individuals (59%) and wealthy traders (34%); the proportion of direct land purchases from the farmers by real estate companies was relatively small (3%). Land sales in Kati are mostly mediated by land speculators (44%) and natives (29%), with less than one-fifth handled by professional surveyors, as shown in Table 3. Table 4 presents the results of the binary logistic model in which various variables were used to predict the probability that a farmer is a victim of land dispossession. The model had a fairly good prediction (% model accuracy = 86%; *χ*^2^ = 50.68, *p* < .001; -*2LogLikelihood* = 65.97; *R*^2^_CS_ = .40, *R*^2^_N_ = .60), with an acceptable level of multicollinearity among the predictors (average VIF = 1.500 and Tolerance > 0.2). Two variables (‘urbanisation adversely affects customary rights’ and ‘other villagers dispossessed of their lands’) positively predicted the likelihood of a respondent being a victim of land despoliation, at 5% significance level. On the other hand, farmers’ level of education, annual farm output and the presence of a land chief in their village decreased the likelihood of being a victim of land dispossession, but at 10% significance level. The likelihood of a farmer being a victim of land despoliation or expropriation could not be confidently predicted by annual income, land registration, size of land owned in the past, and the farmers’ perception of the justice system in land dispute settlement. The bootstrapping technique yielded similar results but increased the certainty levels of the effects of ‘other villagers dispossessed of their land’ (*p* < .001) and ‘annual production’ (*p* < .05) variables, while it indicated that the ‘village has a land chief’ variable was statistically insignificant (*p* > .10).

**Table 4:** Effects of variables on the likelihood of agricultural land loss due to despoilment or expropriation.

Variables	*β*	Wald	*p*-value	Exp(*β*)	95% CI for Exp(*β*)		Colinearity stats.	
					LB	UB	Tolerance	VIF
Family income (in FCFA)	-.102	.455	.549	.903	.670	1.218	.805	1.242
Level of education	-.434*	3.462	.082	1.088	.410	1.023	.543	1.842
Land registration	.571	.458	546	1.770	.339	9.246	.568	1.762
Land size (past)	.452	.286	.119	1.572	.898	2.753	.730	1.370
Urbanisation adversely affects customary rights	3.259**	13.140	.001	26.016	3.866	175.059	.768	1.303
Other villagers dispossessed of their lands	3.145***	9.881	.000	23.229	3.268	165.105	.632	1.583
Village has a land chief	-1.498	3.017	.103	.223	.041	1.023	.951	1.051
Annual production (in tonnes)	-.860**	3.737	.046	.423	.177	1.012	.633	1.580
Perception of justice in land dispute settlement	.515	.518	.450	1.674	.412	6.807	.566	1.768
(Constant)	-2.291	1.664	.205	.101	-	-	-	-

Dependent variable–Victim of land despoliation or expropriation; Model’s accuracy (step 1) = 86.0%, *χ*^2^ = 50.683 (*p* < .001), -2*LL* = 65.968, *R*^2^_CS_ = 0.398, *R*^2^_N_ = .577; Bootstrapped sample size, *N* = 10,000.

***, ** and * denote that the coefficient is significant at 1%, 5% and 10% levels, respectively.

### Effect of urbanisation and decentralisation on agricultural land loss

Urbanisation adversely affects customary rights and consequently increases the likelihood of a farmer being a victim of land despoliation. Additionally, 93% of the respondents confirmed their awareness of the large-scale land sales ongoing in their area, while 75% said there were no lands in the village reserved for future generations. Particularly in Sirakoro Naire village, all the respondents indicated that there was no land available for posterity. Forty-six per cent (46%) of the respondents cited government officials (or the Town Halls) as the sellers.

According to an interview conducted with the General Secretary of the UACDDDD, Massa Kone, this ongoing phenomenon of land grabbing and sales by authorities began with the advent of democracy and decentralisation in 1991 that led to the creation of 701 municipalities throughout the territory. Some newly elected Mayors attack the vital spaces and “lungs of the neighbourhoods” within their jurisdictions (including wetlands, waterways, and areas designated as green spaces) to subdivide them and sell for residential purposes. He also claimed that in some cases, Mayors who have no other resources to finance their political endeavours, or to enrich themselves, target nearby cultivated lands. Moreover, the land transaction activities of the Land Transfer Agency (ACI), since its inception in 2002, have also demonstrated to Malians the value of land, as a good source of income. This observation agrees with a previous report from Bamako [46]. It is, therefore, not surprising that about 30% of the respondents in Kati had sold their lands to earn income to cater for their families. He added that long before, in 1995, titled land became a major form of collateral for accessing credit from banks and mortgages from real estate agencies in the country. However, it is obvious that poor farmers do not enjoy such benefits because they do not have land titles.

### Effect of the justice system and smallholder farmers’ agricultural land loss

Regarding the settlement of land disputes at the law court, over two-thirds (69%) of the farmers were of the view that there is justice in land disputes resolutions. Consequently, farmers’ views did not correlate significantly to land loss through forceful dispossession (Table 4). Some farmers rather blamed the land legislations for their unfairness against the poor because the laws uphold land titles over customary ownership. One respondent observed that “*the first thing a judge asks for as proof of ownership is a land title*, *and failure to produce it means you cannot claim absolute ownership of the land*, *according to the law*. *So*, *you cannot blame the judges*, *but the law*.” Additionally, farmers’ appreciation of the justice system showed a weakly positive but statistically significant correlation (*R* = .309, *p* < 0.01) with their level of education, suggesting that the more educated they were, the higher their appreciation of the legal system.

On the other hand, both leaders of the farmers’ associations and Lawyer Sagara were of a contrary view on the justice system. In many parts of the country, due to poverty, farmers do not have the financial muscles to contend in legal battles individually, but through associations [42]. It is worth mentioning that members of the over two hundred farmers’ associations under the UACDDDD are all victims of land expropriation. Based on this situation, the general secretaries of the UACDDDD and MSV stated that “*the poor are never right in court*,” describing the social inequality smallholder farmers face at the law courts of Mali. Moreover, the leader of the MSV opined that the poor had lost confidence in the justice system because they believe that they lose their cases even before the trials. According to Lawyer Sagara, the plight of farmers in such legal contentions is exacerbated by the unprofessional work done by some judges. He explained that contrary to best practice, some judges pronounce judgments on land disputes without any field verification. Thus, land tenure becomes a policy of exclusion for poor persons.

## Discussion

From this study, the estimated average annual income of the smallholder farmers was far below the national poverty line, indicating that the prevalence of poverty among farmers in Kati is generally high. Although no baseline study on the income of farmers in Kati existed at the time of this study for comparison, it is reasonable to say that Mali’s land tenure reforms have not yielded positive outcomes on rural farmers, as theorized by some experts [4]. Despite these reforms, the farmers have lost significant portions of farmlands, which significantly affect family income. Moreover, our data suggest that larger households have been more affected because the percentage decline in farmland size correlates positively with family size. Agrarian land loss in Kati is chiefly attributed to land sales, despoliation and takeover by the State. Poor smallholders are always the victims of land confiscation by people with political power or the government [47]. Moreover, although the farmers sold their lands chiefly because of despoilment and expropriation threats, land sales were also to buy farm equipment or earn income to cover family expenses. The latter scenario seems to explain why larger families showed a higher percentage decline in farmlands. Additionally, contrary to reports from Thuy Duong, Vietnam, where farmers who lost their farmlands had no vocational training [48], we found no significant correlation between educational level and agricultural land loss in the study area.

Over the years, land title has been and remains the only document that grants ultimate land security in Mali. Titled land is hardly expropriated, and when seized, attracts higher compensation [20]. Previous reforms (i.e. CDF 2000 and LOA 2006) recognised customary land tenure right, which is common in Mali. Yet, they lacked implementation decrees to help formalise such rights to guarantee the security of customary landholdings [1]. In this study, we found that less than one-third of the farmers had registered their lands, and only 6% had full land titles, and this is lower than the 10% reported for rural Sub-Saharan Africa [4]. The other forms of land registrations (i.e. village attestation, award letter and provisional title) are precarious and only offer better security as compared to customary landholdings. Hence, farmers generally have a very low guarantee of land rights and security. Although the results suggest no statistically significant link between land registration and the possibility of land loss through forceful dispossession (Table 4), farmers sold their lands due to land ownership insecurity. Also, we found that the cost per hectare of land sold positively influenced family income, which implies that farmers may increase their income if they get better deals on land sales. However, farmers usually preferred to sell their lands at lower prices than lose them to the State through expropriation. This is partly because although generally, the land laws indicate that the owners of expropriated customary lands must be compensated, evidence of such payments is rare [42].

Clearly, without land titles, smallholder farmers in rural and peri-urban areas will continue to lose their lands, which serve as their means of livelihood. Earlier, Djiré [49] reported an appreciable rise in the rate of land title registration in the Kati *Cercle* between 1984 and 2004 (from 200 to 10,873) but observed that the trend was dissimilar in the rural and peri-urban areas. Yet, only 6% of the farmers surveyed had land titles. This suggests that the challenges encountered by the farmers in acquiring land titles persist despite the reforms over the years. Hence, we discuss the two main barriers the poor farmers in Mali face in securing land titles in the next section.

### Barriers to land security for subsistence farmers

#### Multiple actors, and the complicated and uncoordinated land registration procedure

**Fig 3:**
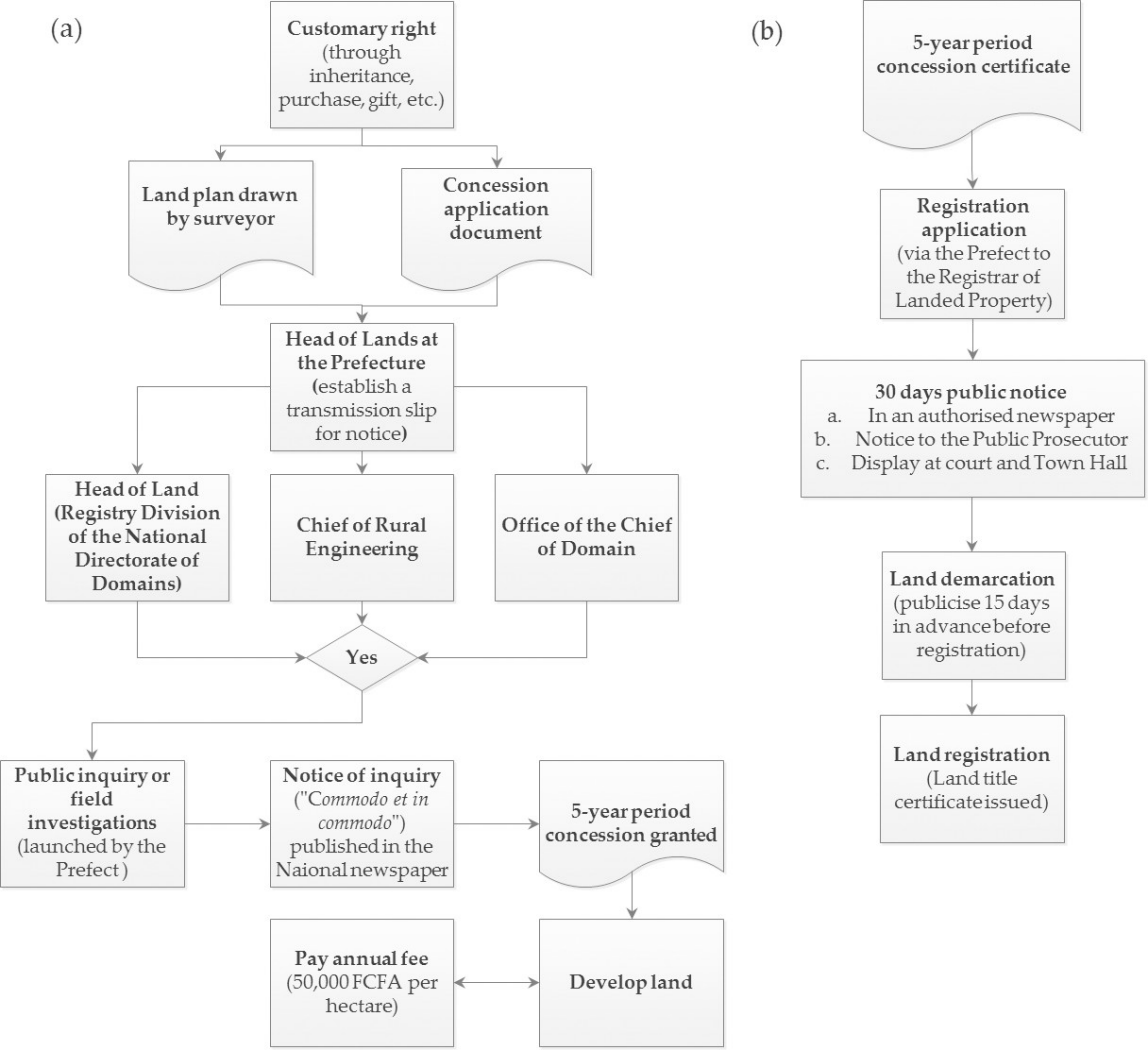
Land registration process in Mali. From (a) customary rights acquisition to rural concession and (b) rural concession to a full land title acquisition. The rural concession process is either terminated or paused until any objection raised by any agent or agency involved is addressed.

The formalisation of customary land rights into a full land title is a two-stage process summarised in Fig 3. The first step is the registration of untitled land (village allocation) as a rural concession (in the name of the State), while the second step transfers the rural concession to the individual whom the land has been assigned, in the form of a land title. A rural concession is granted for five years, within which the holder must satisfy specified conditions, including putting the land to productive use and paying an annual fee of 50,000 FCFA per hectare [49]. To transform the rural concession into a title, the local land commission verifies if the conditions have been satisfied. Additionally, as shown in Fig 3, public inquiry, field investigations and inter-institutional coordination are supposed to play a key role in the process. For example, minutes (*procès verbaux de palabre*, or PVs) of the meetings held during the field investigation must be signed by village authorities. However, they are sometimes ignored by some officials [49].

Land transfers are made at five levels, including the Domain Services, the Governor, the Prefects, the Sub-Prefects and the Mayors, in addition to real estate agencies, and usually without a cadastre. The Domain Services allocate land with titles, either by direct land transfer or the transformation of precarious rights into a definitive title. However, from the survey, the long and complicated procedure is a barrier to most (56%) of the farmers in securing land titles, corroborating an earlier report [49]. Additionally, the results indicated that farmers with a low level of education were less likely to register their lands and more prone to land loss. Some actors and authorities capitalise on farmers’ low educational level and the complex procedures to manipulate the system to dispossess farmers of their lands. According to a key informant, “*on the one hand*, *the Prefects*, *Sub-Prefects*, *Governors and the Mayors keep playing their games*, *while on the other hand the IGM [Geographical Institute of Mali]*, *which is out of the land allocation and registration process*, *often continues to manipulate certain services responsible for land management*.” As summarised by Neimark et al. [42], land conflicts in Mali are typically a tussle between political powers and people knowledgeable in land administration procedures, and the vulnerable.

Additionally, the absence of a cadastre has been well identified as a precursor for land disputes in the country. Therefore, in a state of the nation’s address, delivered in French, on Sept 22, 2013, President Ibrahim Boubacar Keita said, “*Every effort will be made to provide the country with a reliable cadastral system within a reasonable time* [35].” Moreso, Lawyer Sagara added that during the 2009 National Consultation on Land, it was reported that the authorities in charge of managing the land are engaged in various kinds of land speculation but do not face any administrative or criminal sanctions. A recent report corroborated the assertion about the illegal engagements of the authorities in land issues, as 40% of the complaints received by the National Mediator of the Republic between 2017 and 2018, was against public officials [50].

Furthermore, Lawyer Sagara observed that most of the land management problems are usually related to document duplications that purportedly arise from the authority in charge of land management. Duplication is when the land management authority successively allocates the same plot of land to two or more persons. He cited an incident that happened at the Kalaban ACI of the Bamako District, where two persons claimed ownership of a plot of land. One was a resident who possessed an award letter (i.e., a precarious title) dating from 1996, for the plot that he had already developed, whiles the other person had a land title dating from 2011. The land title indicated a direct transfer between the Domain Department and the interested party, with authorisation by the incumbent Governor. However, investigations revealed that there was no direct transfer in 2011, indicating that the title was falsified. Besides, the second land registration was done in the office without cross-checking from other stakeholder agencies or any field investigations, resulting in the document duplication for the same land. Similarly, in Kati, it is reported that the head of Domains and Cadaster noted that about 19,000 land titles were at risk of duplication or falsification [38].

#### High cost of formalisation of customary land rights

The results from Kati show that the farmers are generally poor, and about one-third (31%) of the respondents indicated that they had not registered their lands due to financial constraints. It has been reported that some officials in charge of land title processing demand substantial bribes [47], and it could cost about 2,000–3,000 USD to obtain a full title for a hectare of land [20,49]. On average, this cost is roughly double to triple the annual income of the households surveyed in this study, making it difficult for most farmers to secure land titles. Thus, the current land administration system favours only the rich, who can pay to skip the long queues to get the full titles, corroborating the observation of other scholars that land reforms sometimes result in land “capture by elites” [16]. A recent assessment of Ghana’s LAP also revealed similar challenges of bureaucratic processes and corruption as impediments to the successful implementation of the reform [51]. To assist in this regard, the World Bank has been advocating for institutional streamlining and a reduction of the formalisation fees to promote the conversion of customary rights to titled deeds in Mali [26]. According to the LFA 2017, a certificate can be obtained from the village land commissions and be registered with the municipal authorities or the land administrations, respectively. The law seeks to decentralise land administration to help farmers with a better level of land security at a relatively low cost. However, similar to the case of previous reforms [1,26], there is no decree to enforce its implementation to date. Based on experiences from some Sub-Saharan African countries, some scholars have opined that politicians and government officials are usually reluctant to implement land reforms or do a selective implementation, for fear of losing power over budgets and the land administration workforce [10,52]. Manji [52] observed that in some cases, reform implementation could also be subtly stifled with derisory fund allocation by the government.

As noted earlier, China’s Household Responsibility System (HRS) reform yielded positive outcomes (i.e. boosted agricultural production and farmers’ income) [18], because it properly recognised smallholders and allocated lands to them [17]. Conversely, in Mali, the afore-mentioned land reforms were initiated by the government without proper recognition of the farmers; hence, they have not met the expectations. Therefore, Mali could draw lessons from the Chinese land reform models.

### Commercialisation of agricultural lands and its effects on smallholder farmers

Since the 1980s, international bodies such as the International Monetary Fund (IMF) and the World Bank have been major advocates of land reforms as a driver of economic development in many emerging nations. Before, customary land ownership highly dominated these countries because the local traditions made land inalienable. However, the policies of these institutions have mostly favoured privatisation of land ownership as a means of guaranteeing security and increasing investments in various sectors, including agriculture [10]. Privatisation of land has promoted the commercialisation of land, such as agricultural lands. Moreover, over the years, these organisations have carried out several pro-poor projects to improve land tenure security for farmers [26]. An example is the World Bank-supported National Rural Infrastructure Project (costing USD 11.2 million), which titled 2,400 ha of irrigated areas in the *Office du Nige*r for poor smallholder farmers in 2001–2005. The beneficiaries received titled farmlands after payment (spread over years) of the cost of secondary infrastructure pre-financed by the government; the reimbursed funds was meant for further redevelopment projects.

That notwithstanding, the leader of the UACDDDD partly attributed the problem of farmers losing their lands to the development policies of the IMF and the World Bank. He argued that “*these policies tend to favour the idea that major trading enterprises and multinational companies can better bring development to emergent nations rather than subsistence farming that supports 40% of the food needs in Mali*. *Thus*, *Mali encourages [investments by] multinationals and subsidises the importation of food at the expense of family farming*.” He cited an example that, in 2010, the Ministry of Rural Development and an economic operator, Modibo Keita, signed agreements, which led to the seizure of over 863 hectares of land from the villages of Saou and Sanamadougou, in the *Office du Niger* zone, for intensive potato cultivation. Instead of giving priority to the affected villages which practise family farming to help fight against poverty, the farmers were entirely evicted against their rights to establish a multinational company. His account agrees with earlier reports [47,53]. For example, in the Niger River inner delta, where rice cultivation is practised on land developed and managed by a parastatal institution, *Office du Niger*, farmers are frequently evicted from their lands for non-payment of the so-called water fee. In the 2014–2015 crop year alone, evictions involved 1,592.54 hectares for an amount of 83.2 million FCFA [53]. In such situations, it is the indigent farmers that suffer, as only better-off farmers are able to pay unofficial fees to corrupt officials to retain their lands [47]. Elsewhere in Rwanda, in response to increasing population pressure, soil erosion and the desire for state-backed land security, the government implemented a series of large-scale land and agricultural reforms in 2009 aimed at transforming the rural subsistence production system to a commercial sector of professional farmers [54].

It is worth mentioning that in Mali, the collective management of land is based on customary rights that allow people to live in harmony in society, such that the same land can be used by a farmer whiles pastoralists graze their herds and women for gathering fruits. Thus, the loss of land to commercial operators does not only affect farm owners but other community members who depend on such lands for their livelihoods.

### Justice in land dispute settling for poverty alleviation

A recent report [55] showed that the top land management issues that end up in the law courts of Mali are land grabbing (23%), land ownership/use rights (20%), and boundary disputes (16%), and so on; both disputes over the right of use and expropriation account for 5% each of the problems. Equitable access to justice could, therefore, help poor farmers to secure their lands to sustain their livelihoods and even escape poverty.

Since the majority of the local farmers have no land titles, the ability to defend ownership of their lands, especially in the pre-LFA 2017 era, had been undoubtedly weak at the law courts. Despite this, the majority of the farmers interviewed expressed satisfaction in the justice system regarding the settling of land disputes in the country, while the key informants had contrary views. First, this divergence in opinions could emanate from the respondents’ level of knowledge of the law and how the justice system operates. For example, farmers may not know what judges need to do (e.g. field verifications) before delivering the final decision of the court on land dispute cases. Meanwhile, such unprofessional acts are known and were emphasised in the President’s State of the Nation Address on Sept 22, 2013, when he said, “*There will be an end to land swindling and the spoliation of the poor or the real owners…There will be no more mock trials in the offices of our ethical judges*” [35]. Secondly, the divergence might have emanated from the fact that in Mali, people are generally cautious when publicly expressing their opinions on the justice system. Since most of the respondents were illiterates and, thus, had to be assisted in filling the questionnaires, the presence of a second party likely raised mistrust in the confidentiality level of our data collection process. Hence, some of the farmers probably gave a ‘favourable’ response to the question for security reasons.

### Inequitable land access and security for women and youth

All our respondents were males, and this is not uncommon in many traditional African settings because traditionally, males are mostly the family heads [1,56], and the family head is the custodian of family lands. Even though most of the respondents indicated that women had access to land, it was about access through marriage and parental relationships. Women are, therefore, socio-culturally discriminated against regarding land access in Mali. Furthermore, in the rural areas, the village chiefs give lands to families but hardly to individuals, especially the youth (aged 18–24 years); young individuals are expected to cultivate family lands. They can only inherit land, which will not be their property, or formally rent from the state [47]. Also, as depicted in this study (only 6% of the respondents aged less than 35 years), the youth may be too young to be household heads, and thus qualify as custodians of family lands. Hence, the youth have limited access to lands. Such exclusion generally leads to an increase in poverty in the study area and rural Mali.

To overcome this marginalisation, the LFA 2017 apportions 15% of the agricultural irrigated land to be reserved for vulnerable groups, like women and the youth. Regrettably, to date, this law lacks implementing decrees. Hence, the plight of women and the youth regarding land access and security remains. This situation in Mali seems to mirror the situation in Senegal, where women represent 70% of the rural working population but are particularly affected by land insecurity, as they possess less than 13% of the agricultural land [57]. According to official statistics, women make up about 43% of the agricultural labour force in developing countries. However, they face constraints that reduce their productivity, although they are often a crucial resource in agriculture and the rural economy [58]. Local customs discourage girl-child education, especially in rural areas, and illiteracy facilitates the marginalisation of women because they are mostly ignorant of their rights [42,59]. Thus, women seldom enjoy the right to allocate land, while men use their customary dominance to deprive women of their rights to land. Additionally, women, whose right to cultivate a parcel of land are secured by marital or kinship status, may lose these rights at any time and face reduced access to land; this situation reinforces greater economic and social insecurity for women [60].

### Post-LFA 2017 and other issues

In the past, the government has made efforts to harmonize statutory and customary land laws. Conversely, according to the 2010 Land Tenure Assessment Report [1], it is usually unclear how such reforms will play out practically. In this study, the majority (69%) of the respondents had positive feelings about the LFA 2017. The same proportion affirmed the recognition of customary land rights by the authorities, in recent times, especially after the LFA 2017 was enacted. The General Secretary of the UACDDDD also shared the same opinion. He elaborated that the new law gives legal recognition of the customary land rights by issuing the certificates of landholdings at the community level, which would somewhat represent land titles. In spite of the above, some respondents noted that the law’s impact was yet to be felt in reality, because the situation appears unchanged. For example, the 15% of irrigated agrarian lands allocated to women and the youth, according to the LFA 2017, is not being enforced. It may be argued that it is early days yet to make any conclusions regarding the impact of the LFA 2017 on smallholder farmers. Conversely, since there was no implementing decree to enforce it at the time of writing this paper (3 years after enacting the law), we could only describe it as redundant hope for the poor farmers and the marginalised. Benjaminsen et al. [20] posit that decrees must be adopted without delay to facilitate the implementation of reforms for proper functioning land administration system.

Another issue worth attention pertains to consultations for land reform reviews. According to Lawyer Sagara, the best texts could only be obtained if the opinions of the populations in the far reaches of the Malian ‘bush’ are sought, as much as possible. As some researchers have observed [33], he opined that such people could highlight the real difficulties they face in land management. On the contrary, farmers in very remote areas are mostly side-lined. For example, after the 2009 National Consultation on Lands, an international tender for the review of the CDF 2000 was launched by *Projet d’Appui à la Croissance* (PAC), and the consultancy contract was awarded to a foreign firm. He claimed that the firm ignored farmers in the very remote regions; thus, they missed vital information on the prevailing realities on the ground. To him, such issues have partly culminated in the difficulty of getting permanent solutions to the land problems in Mali. Additionally, after three years of the LFA 2017’s enactment, many land-related debates persist. This demonstrates the need to re-establish spaces for dialogue between actors, especially between farmers’ organisations and the government. According to a recent report [61], the Higher Council of Agriculture, which used to be a privileged space for exchanges, no longer plays this mediation function. Land commissions have problems with representativeness regarding the membership constitution. Furthermore, the village and communal-scale land commission system seems to be poorly adapted to the Sahelian context, particularly regarding pastoral mobility. Thus, it is necessary to integrate pastoral issues into local land management issues as well as the issue of operationalization and the legitimacy of locally negotiated rules in addressing mobility issues.

## Conclusion and recommendations

Over the last two decades, the Malian government has undertaken several reforms to improve land access and security, as a way of reducing poverty. The main reforms since the year 2000 are the CDF 2000, LOA 2006 and LFA 2017. Despite the reforms over the period, this study showed that most of the smallholder farmers in Kati are poor, suggesting that the expected poverty reduction outcomes have not been achieved. Also, the percentage reduction in farmland size and the amount they earned from land sale per hectare significantly influenced farmers’ yearly family income. A review of these reforms indicated that land title remains the only sure way for land tenure security, even though there have been improvements in recognition of customary land rights. Our survey revealed that the process of obtaining a title is long and complicated, and the cost of land title is highly unaffordable to the average farmer. Therefore, only a few farmers have titled lands. We found that there are still institutional lapses in the land administration system, which result in duplication and falsification of land documents. Notably, some government officials are deeply involved in land administration malpractices, such as land speculation and land grabbing, but in almost all cases, they do not face any administrative or criminal sanctions. Furthermore, although the LOA 2006 and, particularly, the LFA 2017 established decentralised institutions for land registration to offer better security for customary landholdings, there are critical barriers to their implementation, such as the lack of implementing decrees and issues with the membership constitution of the land commissions.

In light of this, land insecurity is a big issue and has resulted in a massive loss of farmlands which belonged to the smallholder farmers. In most cases, farmers lose their lands through land sales triggered by either the fear of expropriation or despoliation or as a means to earn income. For fear of losing lands to the State, some farmers rather sell their lands and at lower prices. Hence, the income from such land transactions does not significantly improve their financial status. Also, some of the experts interviewed argued that some development policies of the IMF and the World Bank (e.g., a shift from customary land or state land ownership to private property rights) have adversely affected subsistence farming (which is the source of livelihood for most people in the rural areas), as they promote the commercialisation of agricultural lands. Owing to the above reasons, the prevalence of poverty remains very high among smallholder farmers, despite the reforms.

Based on the review of the reforms and the associated barriers and gaps identified, we recommend that:

- Relevant decrees benchmarking the implementation of pro-poor provisions in land reforms are passed to ensure equitable access and security of land to yield the expected poverty reduction outcomes. In an effort to deter individuals from engaging in unlawful land management practices, the implementation decree should incorporate appropriate administrative and criminal sanctions.
- Institutional reform policies must aim to increase the dialogue among all the stakeholders involved in land management. Also, they must clearly define the authorities of the community institutions and establish appropriate mechanisms for the recognition and registration of collective and individual customary rights.
- Land use planning policies must incorporate a comprehensive land mapping, which demarcates land for specific purposes (e.g. agricultural lands, wetlands, conservation zones, green spaces, etc.).
- Land administration policies should promote a single-institution land administration regime and the digitisation of the land registration using appropriate technologies (e.g. the use of a block-chain system [62,63]) to avoid multiple registration and falsification of land documents which result in land conflicts. More so, measures must be put in place to ensure equitable access to land ownership information on the system. More importantly, this information must be continuously updated and verified to ensure the reliability of the system.

## Supporting information

S1 File Questionnaire administered to the smallholder farmers. (PDF)

S1 Table Definitions of the variables in the multiple linear and logistic regression models. (DOCX)
