# Supplementary material for: Towards poverty alleviation in developing countries: An empirical study of the impact of land tenure reforms in Kati, Mali
Source: PLoS One. 2021 Mar 4;16(3):e0246502. doi: 10.1371/journal.pone.0246502 (PMC7932540; doi:10.1371/journal.pone.0246502)
Supplement: S1 File — (PDF) [file pone.0246502.s001.pdf]

Questionnaire administered to smallholder farmers

This questionnaire is designed to help us assess the current final status of smallholder farmers in Kati and how their source of livelihood are impacted by land tenure reforms in Mali. Your honest views are essential to help us achieve our objective, which could inform future policies on land tenure reforms. Kindly note that all information provided will be held confidential.

Please tick the option that best describes your answer to each of the questions.

Village name:____________________

Demographic information

1. **Age**: Less than 25 ☐ 25-34 ☐ 35-44 ☐ 45-54 ☐ 55 or more ☐
2. **Gender**: Male ☐ Female ☐
3. **Marital status**: Single ☐ Married ☐ Divorced ☐ Widower ☐

4. **What is your level of education?**

None ☐ Adult education ☐ First cycle ☐ Second cycle ☐ Secondary school ☐

Technician diploma ☐ University ☐

5. **Occupation:** Agriculture only ☐ Public service ☐ Private employee

Self-employed ☐ Other ☐

6. **How many people are in your family?**

Less than 5 ☐ 5-10 ☐ 11-16 ☐ 17 or more ☐

7. **What is your family's annual income in CFA Franc?**

Less 200,000 ☐ 200,000-299,999 ☐ 300,000- 399,999 ☐ 400, 000- 499,999 ☐

500,000-599,999 ☐ 600, 000-699,999 ☐ 700,000-799,999 ☐ 8.800,000-899,999 ☐

900,000-999,999 ☐ 1,000,000 or more ☐

Land access and security and agricultural productivity

8. **How did you access your land(s)?**

Intra lineage access ☐ Gift ☐ Lend ☐ Town Council ☐ Purchase ☐

Rent ☐ Other ☐

9. **What type of land tenure system applies to your land(s)?**

Customary ☐ Modern ☐ Mixed ☐

10. **Is there a chief of land in your village?**

Yes ☐ No ☐

11. **Do women have access to land?**

Yes ☐ No ☐

12. **Does urban expansion affect the customary regime?**

Yes ☐ No ☐

13. **Are customary land rights recognised by the government authorities?**

Yes ☐ No ☐

14. **How do you appreciate modern land management law (*Loi d'Orientation Agricole*)?**

Bad ☐ Good ☐

15. **Does land tenure dualism alleviate poverty?**

Yes ☐ No ☐

16. **Have you registered or secured your land(s)?**

Yes ☐ No ☐

17. **If yes (for Q16), what type of land security do you have?**

Provisional title ☐ Land title ☐ Award letter ☐ Village Attestation ☐

18. **If no (for Q16), why have you not registered your land?**

Lack of financial resources ☐ Long and complicated registration procedure ☐

Other ☐ ____________________(*please state*) Not sure ☐

19. **Why did you sell a part of your land?**

Family needs ☐ Fear of losing to the government ☐ To purchase equipment ☐

Other ☐

20. **Have you been a victim of land despoliation or expropriation?**

Yes ☐ No ☐

21. **Have some villagers been dispossessed of the lands forcefully?**

Yes ☐ No ☐

22. **Is there justice in the settlement of land disputes?**

Yes ☐ No ☐

23. **Are local people aware of their large-scale land sales?**

Yes ☐ No ☐

24. **To whom are the lands sold?**

Government ☐ Real estate companies ☐ Rich traders ☐ Individuals ☐

More than one option ☐

25. **For what purpose do they buy the land?**

Housing ☐ Agriculture ☐ Breeding ☐ Land speculation ☐ Other ☐

26. **How do sellers come into contact with a land buyer?**

Through an agent ☐ Professional surveyor ☐ Bailiff ☐ Land speculator ☐

Native ☐ More than one option ☐

27. **How much do you sell 1 hectare of land in CFA franc?**

Less than 200,000 ☐ 200,000 – 299,999 ☐ 300,000- 399,999 ☐

400,000- 499,999 ☐ 500,000 or more ☐

28. **Is there any land reserved for the future generation in your village?**

Yes ☐ No ☐

29. **What is the size of your land currently (in hectares)?**

Less than 2 ☐ 2-5. 99 ☐ 6-9.99 ☐ 10-13.99 ☐ 14 or more ☐

30. **What was the size of your land in the past (in hectares)?**

Less than 2 ☐ 2-5. 99 ☐ 6-9.99 ☐ 10-13.99 ☐ 14 or more ☐

31. **By about what percentage has the size of your farmland declined?**

Less than 20 ☐ 20-39 ☐ 40-59 ☐ 60-79 ☐ 80 or more ☐

32. **What is your production all cereals combined annually (bags of 100 kg)?**

20-39 ☐ 40-59 ☐ 60-79 ☐ 80-99 ☐ 100-119 ☐ 120-139 ☐

140-159 ☐ 160-179 ☐ 180- 199 ☐ 200 and more ☐
