# Supplementary material for: Towards poverty alleviation in developing countries: An empirical study of the impact of land tenure reforms in Kati, Mali
Source: PLoS One. 2021 Mar 4;16(3):e0246502. doi: 10.1371/journal.pone.0246502 (PMC7932540; doi:10.1371/journal.pone.0246502)
Supplement: S1 Table — (DOCX) [file pone.0246502.s002.docx]

**S1 Table.** **Definitions of the variables in the multiple linear and logistic regression models.**

| **Variable** | **Unit** | **Category (Code)** |
| --- | --- | --- |
| Age | yr | <25 (1); 25–34 (2); 35-44 (3); 45–54 (4); ≥55 (5) |
| Occupation |  | Farming only (0); farming plus other (1) |
| Level of education |  | None (0); adult education (1); 1^st^ cycle (2); 2^nd^ cycle (3); secondary (4); diploma (5); university (6) |
| Family size | person | <5 (0); 5–10 = 1; 11–16 = 2; >16 (3) |
| Land size (past/present) | ha | <2 (1); 2–5.99 (2); 6–9.99 (3); 10–13.99 (4); ≥14 (5) |
| % decline in farm size | % | <20 (1); 20–39 (2); 40–59 (3); 60–79 (4); ≥80 (5) |
| Annual income | FCFA | < 200,000 (1); 200,000–399,999 (2); 400,000–599,999 (3); 600,000–799,999 (4); 800,000–999,999 (5); ≥ 1,000,000 (6) |
| Income from land sale | FCFA/ha | <200,000 (1); 200,000- 299,999 (2); 300,000–399,999 (3); 400,000–499,999 (4); ≥ 500,000 (5) |
| Annual farm output | tonnes | < 2.3 (1); 2.3–4.5 (2); 4.6–6.8 (3); 6.9–9.1 (4); ≥ 9.2 (5) |
| Land registration |  | No (0); yes (1) |
| Urbanisation adversely affects customary rights |  | No (0); yes (1) |
| Other villagers dispossessed of their lands |  | No (0); yes (1) |
| Village has a land chief |  | No (0); yes (1) |
| Perception of justice in land dispute settlement |  | Bad (0); Good (1) |
| Victim of despoliation or expropriation |  | No (0); yes (1) |
